# Excitatory drive of masseter muscle during mastication with dental implants

**DOI:** 10.1038/s41598-018-26926-z

**Published:** 2018-06-05

**Authors:** Anastasios Grigoriadis, Mats Trulsson

**Affiliations:** Section of Oral Rehabilitation, Department of Dental Medicine, Karolinska Institutet, Huddinge, Sweden

## Abstract

Previously we have reported a biphasic increase in excitatory drive of the masseter muscle during natural chewing in young adults. We now hypothesize that sensory inputs from the periodontal mechanoreceptors (PMRs) are responsible for the late increase in excitatory drive during this biphasic movement. 13 participants with implant-supported bridges in both jaws, and thus lacking PMRs, and 13 participants with natural dentition chewed and swallowed model food of different hardness. Electromyographic (EMG) activity of the masseter muscle was recorded, along with the position of the mandible, and the muscle activity and jaw kinematics during the different phases of the chewing cycle were analyzed. Throughout the entire masticatory sequence, the excitatory drive of the masseter muscle during the jaw closing increased in a biphasic manner for the dentate participants; whereas biphasic elevation was observed only during the middle and last segments in the implant participants. Dentate participants exhibited significantly greater boosting of the EMG activity during late jaw closing than the implant participants, irrespective of food hardness and segment of the masticatory sequence. Sensory information from PMRs are required for boosting the enhancement of masseter muscle activity during the late jaw closing, during tooth-food contact.

## Introduction

During mastication, the central pattern generators in the brain stem continuously senses inputs, primarily from the muscle spindles in the jaw-closing muscles and from the periodontal mechanoreceptors (PMRs)^1,2^. These inputs contribute to the adaptation of jaw muscle activity to the mechanical properties of the food^1–9^. In addition to the muscle activation required for jaw closing movements during chewing, muscle activity is also required to overcome the resistance of the food. Studies on humans performing simulated chewing movements have shown that the parameters of this **“**additional muscle activity” (AMA) are partially set in advance on the basis of food resistance predicted from sensory experiences during preceding chewing cycles and partially modulated in immediate response to direct feedback from oral mechanoreceptors^10,11^. Experiments on anaesthetized rabbits indicate that a component of AMA that can precede tooth-food contact is unaffected by blocking the PMRs, but abolished by inhibiting the muscle spindles^12,13^. Likewise, other animal studies suggest that signals from muscle spindles are most important during the early phase, whereas inputs from both muscle spindles and PMRs are important during the later phases of force generation^12–16^.

During natural chewing in humans, the activity of the jaw muscles is greater when chewing hard than soft food and gradually decreases during the masticatory sequence^3,5,17^. Individuals with implant-supported bridges, and thus lacking PMRs, show impaired adaptation of muscle activity to food hardness^3^, indicating an important role for sensory signals from PMRs in this connection. More specifically, individuals with implants exhibit a significantly weaker elevation in EMG activity early during the masticatory sequence as food hardness increases than individuals with natural dentition. In addition, the reduction in muscle activity during the progression of the masticatory sequence is substantially lower in individuals with implants.

In an earlier examination of variations in the masseter muscle activation with time during natural chewing cycles in young adults, we found that the excitatory drive of the masseter muscle during the jaw-closing phase increases in a biphasic manner showing an early and a late component^17^. Since the transition between these components occurs approximately at the time of tooth–food contact, we proposed that signals from PMRs during the initial tooth-food contact contribute to initiation of the late component. Therefore, we tested this hypothesis by analyzing the EMG activity of the masseter muscle during the jaw-closing phase in individuals with dental implants chewing food items of different hardness. We predicted that since these individuals lack PMRs, the development of EMG activity following tooth-food contact is disturbed in comparison to dentate individuals. Furthermore, since the PMRs are particularly suited to convey detailed information about contact between the food and dentition during biting and chewing^8,18–24^ we predicted that modulation during the early component of the masticatory sequence in response to changes in the properties of food might be impaired in individuals with implants. If so, this would suggest that the PMRs are critical for obtaining information concerning the current properties of the food for use in subsequent chewing cycles.

## Materials and Methods

### Participants

The study involved thirteen participants with natural dentition including at least 28 permanent teeth and thirteen participants with implant-supported bridges in both the jaws for at least twelve months. Each group consisted of 5 women and 8 men each with mean ages of 71.1 years (range 58–83) and 66.4 years (range 59–79), respectively. None of the participants indicated any problems or dysfunction associated with chewing and all stated that they ate comfortably. The study was conducted in accordance with the guidelines set forth in the Declaration of Helsinki II and approved by the regional ethical review board in Stockholm, Sweden. Informed consent was obtained from all participants before the start of the experiment.

### Apparatus and experimental protocol

**Figure 1:**
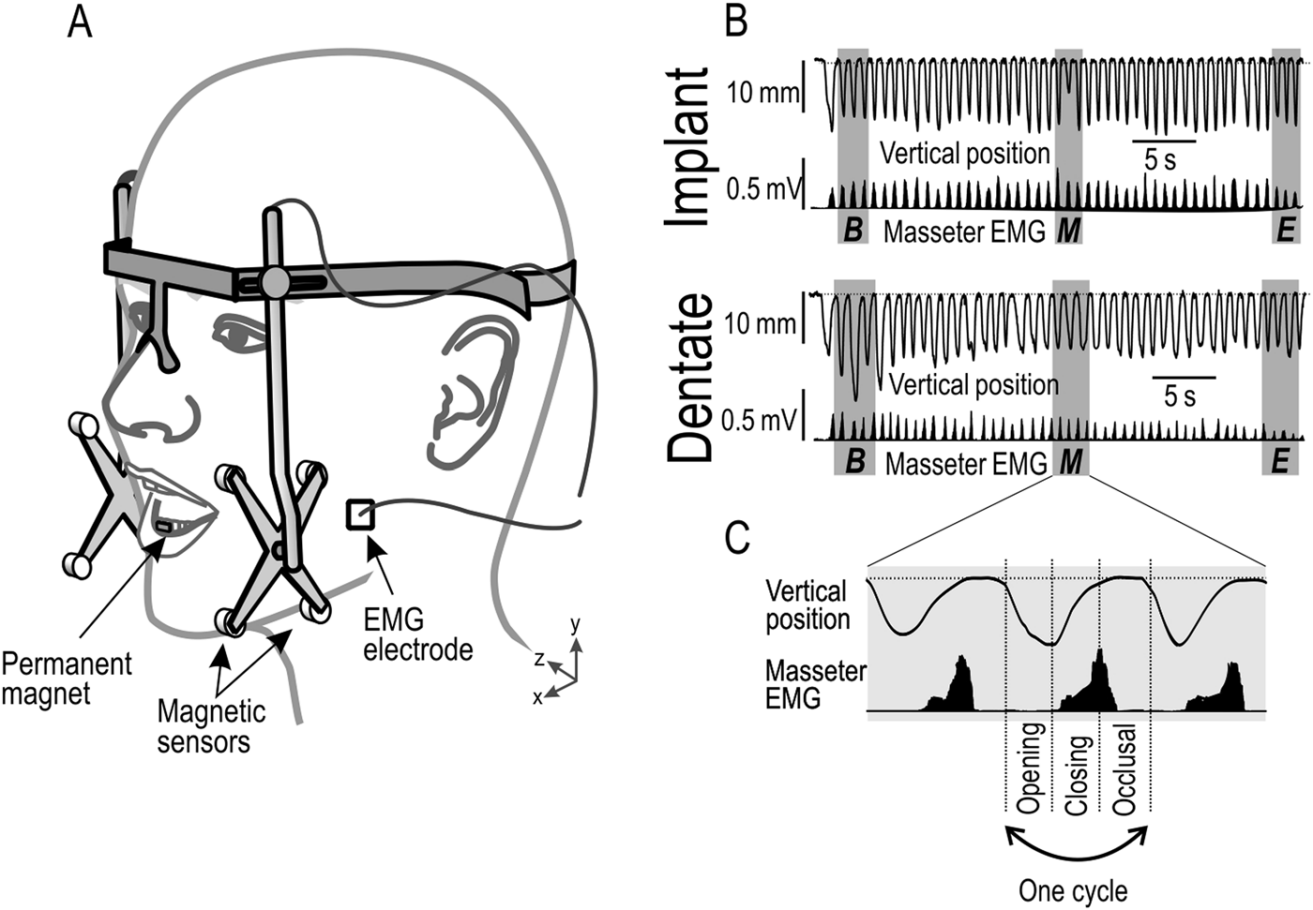
Apparatus, recorded signals and phases of chewing cycles. (**A**) Electromyographic signals (EMG) from the masseter muscle were recorded by bipolar surface electrodes. Jaw movements were monitored by tracking the position of a small magnet attached to the labial surfaces of the lower incisors, with magnetic sensors in a lightweight frame mounted on the head. (**B**) Vertical position of the mandible and EMG activity (r.m.s. processed) during representative masticatory sequences, performed by a dentate and an implant participant. The grey boxes indicate the beginning (***B***), middle (***M***) and end (***E***) of this sequence. (**C**) Expansion of the middle segment in (**B**) illustrating the opening, closing and occlusal phases of one cycle.

The apparatus and general experimental procedures have been described in detail previously (Grigoriadis *et al*.^3,14^) and has also been used in previous studies^25,26^. In brief, each participant chewed and swallowed four soft and four hard visco-elastic standard items of food while the movements of the lower jaw were monitored in three dimensions by a lightweight, head-mounted, custom-made tracker (accuracy: 0.1 mm; bandwidth: DC – 100 Hz) (Fig. 1A). This apparatus allowed normal head movements while chewing. The gelatin-based food items were cylindrical in shape (diameter = 20 mm; height = 10 mm) and presented in random order. Surface EMG signals were recorded from the center of the masseter muscle on the preferred chewing side using bipolar electrodes (2 mm in diameter and 12 mm apart; bandwidth 6 Hz to 2.5 kHz).

The participants were given verbal instructions to first hold the test food between the tongue and palate with the teeth in the inter-cuspal position (reference point for the kinematic analysis) for a few seconds before starting to chew. Once done with chewing and swallowing, the participants were instructed to return to the inter-cuspal position.

### Data analysis

We analyzed the beginning, middle and end of each masticatory sequence (Fig. 1B). For each of these segments data from three consecutive cycles were averaged. A chewing cycle was defined as consisting of (i) an opening phase beginning when the jaw was opened 1 mm from the occlusal state and ending at peak jaw opening; (ii) a closing phase that ended when the jaw returned to the same vertical position at which the opening phase began; and (iii) an occlusal phase that started at the end of the closing phase and ended when the opening phase of the subsequent chewing cycle began (Fig. 1C).

The EMG signal (sampled at 3.2 kHz), was processed by root mean square (r.m.s.) across a moving time window of ±31 ms. To make the EMG data of the different participants comparable, we normalized the time-varying signal by dividing it by the mean activity during all chewing cycles for each participant. To preserve temporal information, for each cycle we normalized each phase to the mean duration of that same phase during all cycles for all participants in each group (see Grigoriadis *et al*.^14^). We empirically defined the transition between the early and late components of the increase in the excitatory drive of the masseter muscle during the jaw-closing phase as the first point after the start of this phase at which the second derivative of the normalized EMG signal first reached 300 normalized EMG units/s^2^ (NU/s^2^).

We assessed the differences between the two groups of participants (dentate and with implants) using mixed-design, repeated measures ANOVAs. All ANOVAs were based on mean values computed for each participant and combination of factorial levels. A P-value < 0.05 was considered statistically significant.

## Results

All subjects were able to perform the chewing sequence as instructed. Since quantitative parameters of jaw-movements and integrated EMG activity during chewing cycles have been described earlier for these same dentate and implant participants (see Grigoriadis *et al*.^3^), the present focus will be mainly on a comparison of these groups with respect to the variation in activation of the masseter muscle with time.

**Figure 2:**
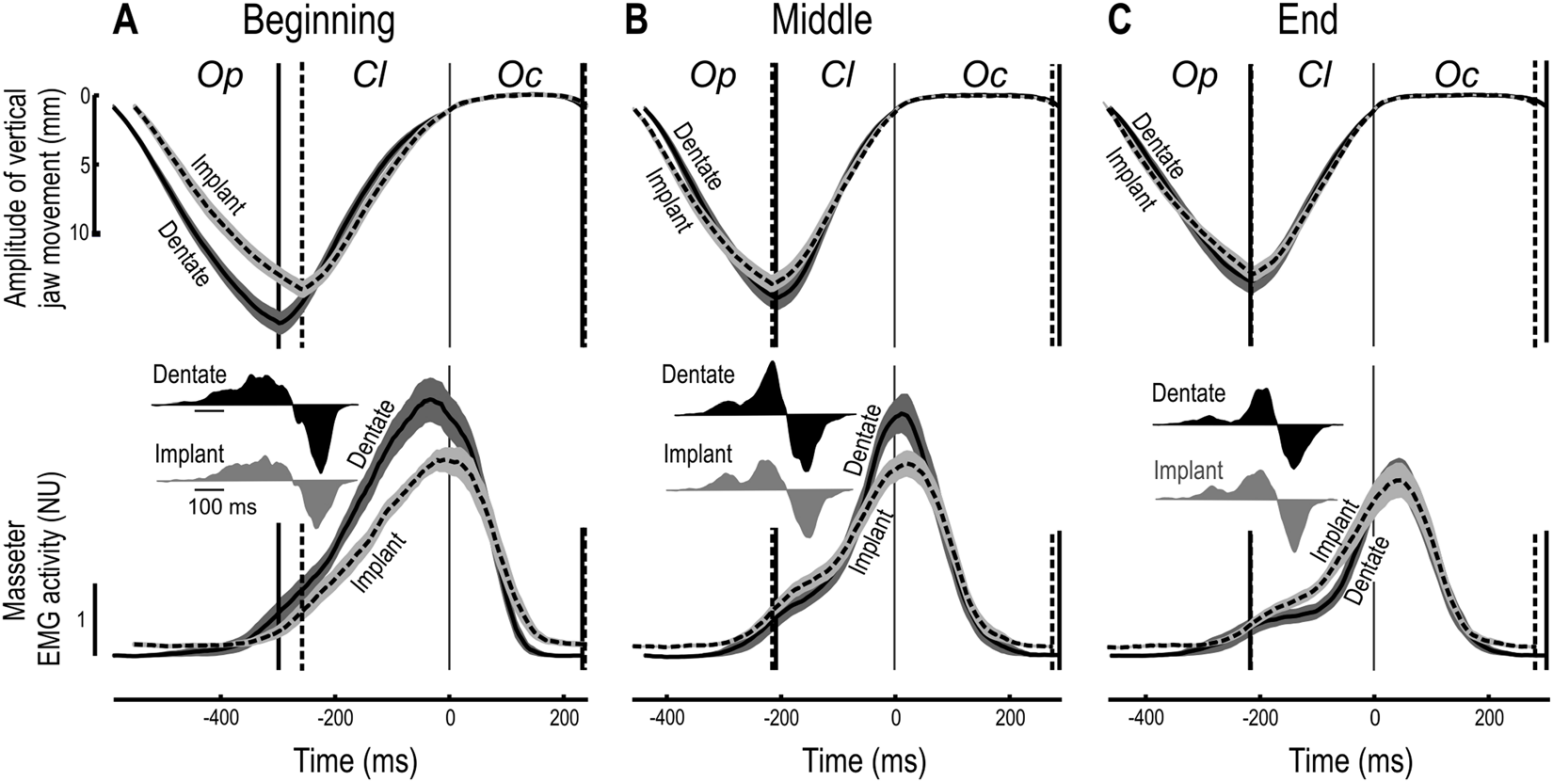
Vertical jaw movement and normalized masseter EMG activity (NU) for the dentate (solid curves) and implant participants (dashed curves) during single cycles of chewing on hard food. Data shown for the beginning (**A**), middle (**B**) and end (**C**) of the masticatory sequence are the averages for all participants and chewing cycles in each group (following normalization of each phase to the mean duration of that same phase for all participants in each group). Grey areas indicate SE (N = 13) and the data have been aligned temporally to the start of the occlusal phase (time = 0). The vertical lines to the left and right indicate the start of the closing phase (peak jaw-opening) and end of the occlusal phase, respectively. *Op*, *Cl* and *Oc* indicate the jaw-opening, jaw-closing and occlusal phases, respectively. The inset illustrates the rate of change in the average EMG signal.

Regarding the hard food Fig. 2 compares the changes in vertical jaw movements and masseter EMG activity during the masticatory sequence for the dentate and implants participants. As the food was particularized, the masseter EMG activity (integrated across the entire chewing cycle) gradually declined as the masticatory sequence progressed (F_2:48_ = 30.5; p < 0.001), Furthermore, this modulation of the muscle activity was significantly smaller in the implant group than in the dentate group (F_2:48_ = 6.0, P < 0.01 for a two-way interaction between the segment of the masticatory sequence and group). (For details regarding changes in integrated EMG activity during the different phases of the chewing cycle, see Grigoriadis *et al*.^3^).

As the masticatory sequence proceeded, the peak EMG signals were also reduced (F_2:48_ = 8.3; p < 0.001, main effect segment) to a greater extent for the dentate group (F_2:48_ = 3.6; p < 0.05, two-way interaction between segment and group). Overall, the time of peak EMG activity relative to the onset of the occlusal phase shifted gradually during the sequence (F_2:48_ = 34.5; p < 0.0001, main effect segment), with no significant difference between the two groups in this respect (no main effect of group and no interaction that involved group). In the beginning, this peak preceded the onset of the occlusal phase by approximately 24 ± 4.6 ms (dentate; mean ± SD, n = 13) and 17 ± 4.4 ms (implant, n = 13); whereas in the middle segment it lagged behind the onset by approximately 16 ± 2.8 ms and 26 ± 3.5 ms, respectively. At the end of the sequence, the peak EMG activity was observed 40 ± 2.9 ms and 36 ± 8.9 ms in the dentate and implant groups, respectively, following the onset of occlusion (p > 0.05 between groups in each segment).

As previously shown for healthy young adults (Grigoriadis *et al*.^14^), the increase in the excitatory drive of the masseter muscle during jaw closing in the dentate group was biphasic, with an early and a late component throughout the masticatory sequence (see the insets in Fig. 2, black curves). However, for the implant group there were no clear signs of a biphasic muscle drive in the beginning of the masticatory sequence (Fig. 2A), but only during the middle and final segments (Fig. 2B and C). For the dentate group, the estimated point of transition between the early and the late components changed for the masticatory sequence (F_2:24_ = 32.6; p < 0.001). During the first segment of this sequence, it occurred when the jaw was approximately 12 mm open, which corresponded approximately to the size of the food items. The corresponding values for the middle and end of the masticatory sequence were 9.5 and 7.2 mm, respectively. Occurrence of this transition at significantly smaller jaw openings as the size of the food particles was gradually reduced during the masticatory sequence suggests that the transition took place at about the time of food contact (see further Grigoriadis *et al*.^14^). With respect to the middle and end segments of the masticatory sequence (for which data were available for both groups), a main effect of segment (F_1:24_ = 79.7; p < 0.001), but also a two-way interaction between segment and group (F_1:24_ = 14.9; p < 0.001) was observed. This interaction indicated that the reduction in jaw opening at the point of transition between the early and late components of the masticatory sequence was less for the implant (8.9 mm in the middle and 8.0 mm at the end) than for the dentate group (9.5 and 7.2 mm).

**Figure 3:**
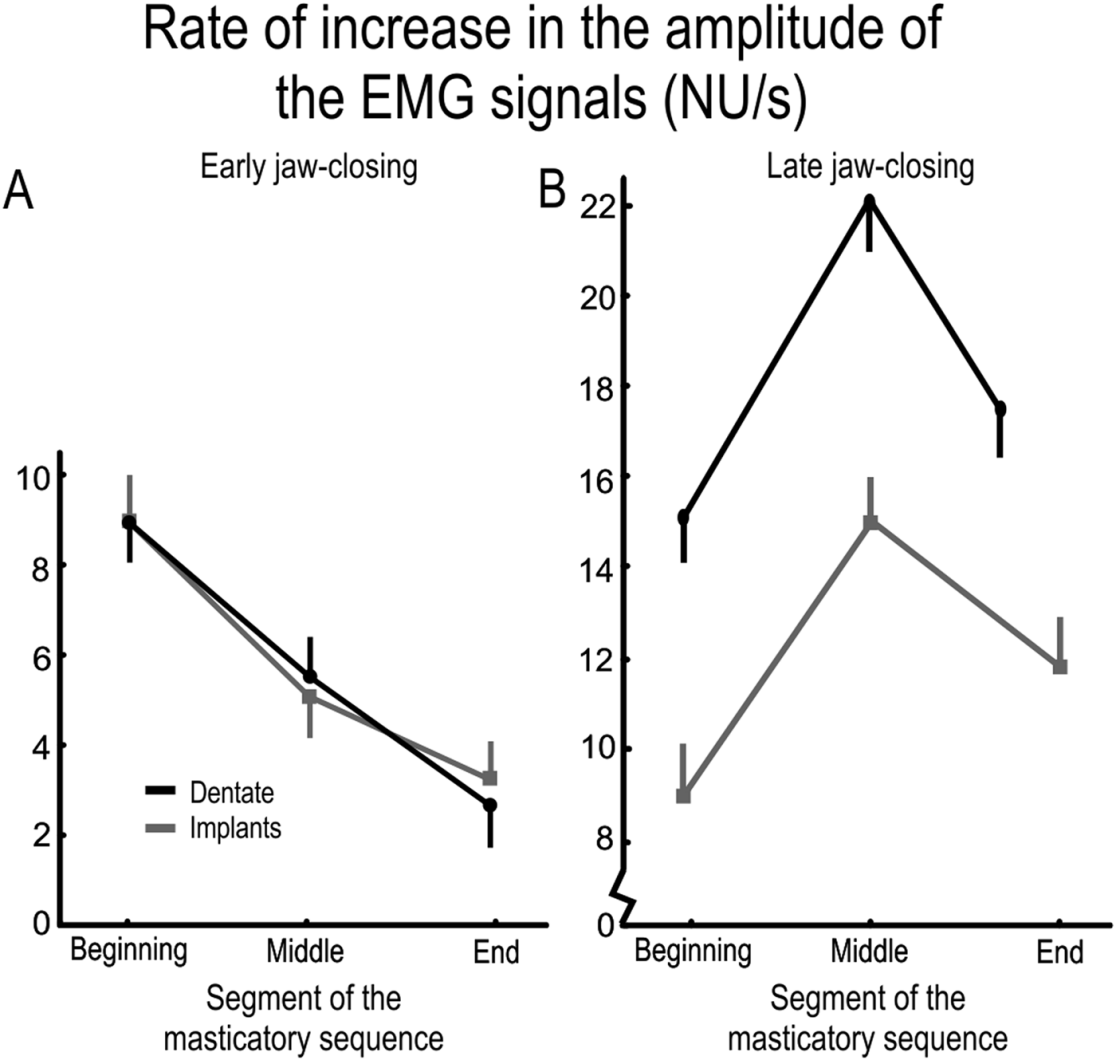
Rate of increase in the amplitude of the EMG signals (NU/s) during (**A**) early jaw-closing (80 ms after the beginning of the jaw-closing phase) and (**B**) late jaw closing (180 ms after the beginning of the jaw closing phase, for chewing cycles in the beginning, middle and end of the masticatory sequence. The symbols depict the mean values and the bars show SEM (in one direction) for the dentate (black; n = 13) and implant participants (grey; n = 13).

As a measure of adaptation of muscle activity prior to tooth-food contact during the masticatory sequence, the average rate of increase in EMG activity during the early jaw closing phase (i.e., 80 ms after the beginning of the jaws closing phase) was computed and found to decrease during the masticatory sequence (F_2:48_ = 58.5, p < 0.001; main effect segment). Notably, there was no difference between the two groups in this respect (Fig. 3A). In contrast, the maximal rate of increase in the EMG activity, which occurred later during the late jaw closing phase (i.e.,180 ms from the beginning of the jaw closing phase), did differ between the groups (F_1:24_ = 25.7; p < 0.001; Fig. 3B). Regardless of the food hardness and segment of the masticatory sequence, the dentate participants demonstrated significantly stronger boosting of the EMG activity during the late component than those with implants (F_2:48_ = 58.5, p < 0.001; Fig. 3B).

**Figure 4:**
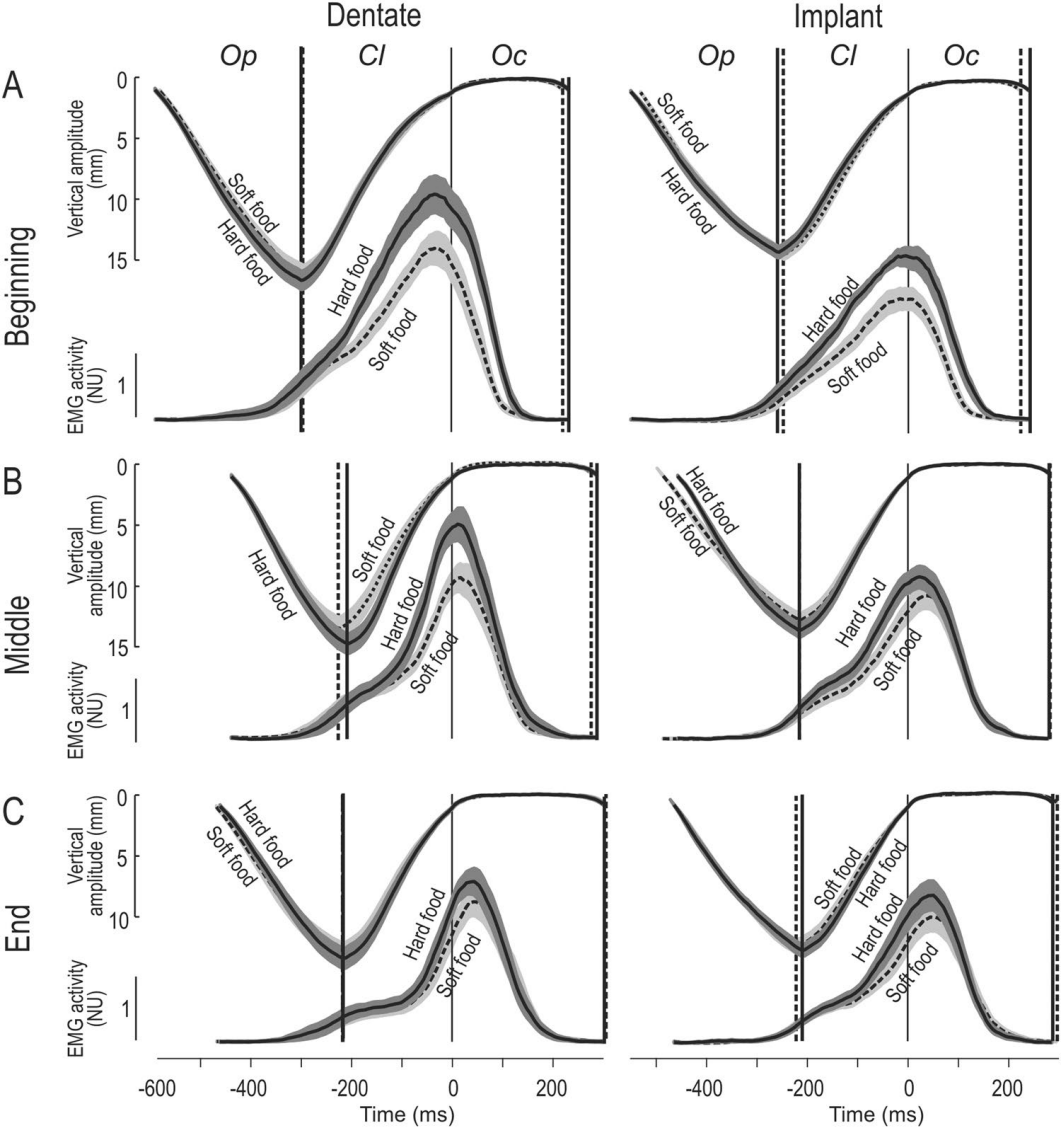
Vertical jaw movement and masseter EMG activity when the dentate and implant participants were chewing hard (solid curves) or soft (dashed curves) food during the beginning (**A**), middle (**B**) and end (**C**) of the masticatory sequence. For further details, see the legend to Fig. 2.

Figure 4 illustrates the influence of food hardness on the temporal profile of masseter muscle activation for the dentate and implant participants. Overall, chewing soft food was associated with lower EMG activity (integrated over the entire chewing cycle) than chewing hard food (main effect of food type; F_1:24_ = 42.6; p < 0.0001) and, as described above, the activity declined during the masticatory sequence (F_2:48_ = 14.6; p < 0.0001), to a greater extent with hard than soft food (interaction between food type and segment; F2:48 = 5.6; p < 0.01; Fig. 4). In addition, our data suggest that this decline in masseter EMG activity was less pronounced in the implant than in the dentate group (interaction between group and segment; F_2:48_ = 4.9; p < 0.05).

**Figure 5:**
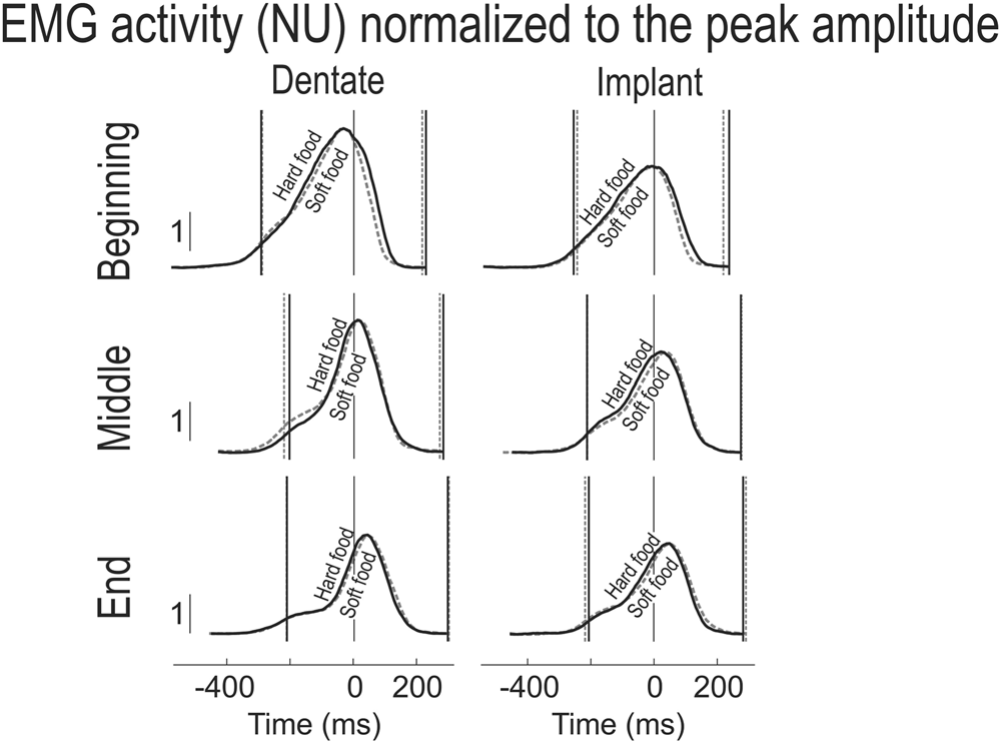
Masseter EMG activity when the dentate and implant participants were chewing hard (solid curves) or soft (dashed curves) food. The same average muscle activity as in 4A–4C, but normalized to the peak amplitude for each group. For further details, see the legend to Fig. 2.

For both groups of participants, our findings indicate that the scaling of EMG amplitude during the masticatory sequence to the properties of the food was in general proportional, resulting in little change in the time-course of muscle activation. In other words, after normalization of the variation in muscle activity with time to peak amplitude for each group and segment of the masticatory sequence, the temporal profiles for hard and soft food were similar (Fig. 5). Finally, with dentate participants chewing soft food an early and a late component of the increase in EMG activity during the jaw-closing phase could also be discerned throughout the masticatory sequence; whereas, in the case of the implant participants chewing soft food, this biphasic pattern was present only in the middle and end of the sequence (Figs 4B and 4C).

## Discussion

Here, we compared the temporal profile of masseter muscle activity in individuals with natural dentition or implant-supported bridges in both jaws during chewing cycles involving hard or softer food. One major finding was that throughout the masticatory sequence the dentate participants demonstrated a biphasic muscle drive during the jaw-closing phase, as previously shown for young adults^17^; whereas, those with implants did not exhibit a clear biphasic muscle drive in the beginning of the masticatory sequence. In other words, the implant participants, who lacked periodontal mechanoreceptors (PMRs), failed to boost the increase in muscle activity at the end of the jaw-closing phase early during mastication. Indeed, as reported previously, such individuals have difficulty in regulating the bite forces^27,28^ and biting through food early in the sequence (Grigoriadis^3^). The lack of a distinct late component of muscle drive in the absence of PMRs suggests that inputs from these receptors upon tooth-food contact play an important role in boosting the muscle drive to overcome food resistance. This interpretation is in agreement with reports that blocking information from the PMRs of anaesthetized rabbits significantly reduces the masticatory force during the “power phase” of the chewing cycles^15,16^.

It is noteworthy that, despite their lack of a biphasic increase in the excitatory drive of the masseter muscle during jaw closing in the beginning of the masticatory sequence, the participants with implants showed a biphasic increase later in this sequence. Most likely, the information concerning food properties acquired during preceding cycles of chewing, in combination with a slow change in these properties in successive cycles, allowed predictive adaptation. In other words, commands were presumably sent to the muscles in advance, allowing them to adapt on the basis of sensory cues acquired during preceding chewing cycles. In the absence of PMRs, the most important sensory information presumably originated from muscle spindles in the muscles that close the jaw^29^. Indeed, studies on animals have suggested that signals from muscle spindles are critical for various facilitatory masseteric responses guided by a feed-forward mechanism based on information from previous chewing cycles^12,13^.

Our finding that individuals with implants adapt muscle activity prior to the onset of tooth-food contact during the masticatory sequence in a fashion that apparently matches alterations in the properties of the food, indicates that information from the PMRs is not necessary for regulation of muscle activity before tooth-food contact. This conclusion corroborates earlier findings in anaesthetized rabbits that the AMA prior to food contact is unaffected by blocking the PMRs, but eliminated by impairing input from the muscle spindles^12^. In essence, in our implant participants most adaptation of the EMG activity during the jaw-closing phase of the masticatory sequence observed appears to result from scaling of the rate of increase in EMG before the tooth-food contact. In contrast, the more robust and stronger adaptation in dentate participants seems to reflect additional scaling of the late, post-contact component, presumably in response to signals from the PMRs. Further, the adaptation of mastication to food hardness observed in both the dentate (see also Grigoriadis *et al*.^14^) and implant participants, involving proportional scaling of EMG amplitude without appreciable alteration of the temporal profile of the muscle activation, indicates that PMRs are not required for such scaling. These findings are also in accordance with previous study that show inferior neuromuscular coordination during chewing in people with dental implant compared to dentate participants^30^.

In conclusion, lack of signals from PMRs in individuals with implant-supported bridges reduces adaptation of muscle activity during the masticatory sequence, since boosting of the activity of the muscles that close the jaw during tooth-food contact is impaired and, indeed virtually absent during the initial part of the sequence. The remaining adaptation of muscle activation in implant individuals to changes on food properties appears to depend largely on regulation of muscle activity prior to tooth-food contact, which occurs in the absence of signals from PMRs. Feedback concerning food contact provided by the PMRs appears to be most critical in the beginning of the masticatory sequence, when the food is initially encountered; whereas prediction of food properties based on other mechanoreceptor systems can be utilized more effectively later during the sequence, when the properties of the bolus only change gradually with each successive chewing cycle and are therefore more “familiar”.

The current study may have clinical implications in planning future clinical procedures and research studies. Oral rehabilitation procedures generally aims at restoring the normal occlusion and lost anatomical structures without particular emphasis on restoring chewing function. However, the results from the current study indicates that mere restoring the anatomy of the oral structures to manage edentulism does not necessarily guarantee automatic improvement of patient’s chewing function. Chewing problems associated with contemporary prosthodontic means are perhaps due to poor retention of the prosthesis or impaired sensorimotor regulation. While the current treatment procedures with dental implant have obviously improved the prosthesis retention, impaired sensorimotor regulation may still persists.
